# PRL-3, a Metastasis Associated Tyrosine Phosphatase, Is Involved in FLT3-ITD Signaling and Implicated in Anti-AML Therapy

**DOI:** 10.1371/journal.pone.0019798

**Published:** 2011-05-12

**Authors:** Jianbiao Zhou, Chonglei Bi, Wee-Joo Chng, Lip-Lee Cheong, Shaw-Cheng Liu, Sylvia Mahara, Kian-Ghee Tay, Qi Zeng, Jie Li, Ke Guo, Cheng Peow Bobby Tan, Hanry Yu, Daniel H. Albert, Chien-Shing Chen

**Affiliations:** 1 Cancer Science Institute of Singapore, National University of Singapore, Singapore, Singapore; 2 Department of Medicine, Yong Loo Lin School of Medicine, National University of Singapore, Singapore, Singapore; 3 Department of Hematology-Oncology, National University Hospital, Singapore, Singapore; 4 Institute of Molecular and Cell Biology (IMCB), A*Star, Biopolis, Singapore, Singapore; 5 Department of Physiology, Yong Loo Lin School of Medicine, National University of Singapore, Singapore, Singapore; 6 Institute of Biotechnology and Nanotechnology, A*Star, Biopolis, Singapore, Singapore; 7 Abbott Laboratories, Chicago, Illinois, United States of America; 8 Division of Hematology and Oncology, School of Medicine, Loma Linda University, Loma Linda, California, United States of America; The National Institute of Diabetes and Digestive and Kidney Diseases, United States of America

## Abstract

Combination with other small molecule drugs represents a promising strategy to improve therapeutic efficacy of FLT3 inhibitors in the clinic. We demonstrated that combining ABT-869, a FLT3 inhibitor, with SAHA, a HDAC inhibitor, led to synergistic killing of the AML cells with FLT3 mutations and suppression of colony formation. We identified a core gene signature that is uniquely induced by the combination treatment in 2 different leukemia cell lines. Among these, we showed that downregulation of PTP4A3 (PRL-3) played a role in this synergism. PRL-3 is downstream of FLT3 signaling and ectopic expression of PRL-3 conferred therapeutic resistance through upregulation of STAT (signal transducers and activators of transcription) pathway activity and anti-apoptotic Mcl-1 protein. PRL-3 interacts with HDAC4 and SAHA downregulates PRL-3 via a proteasome dependent pathway. In addition, PRL-3 protein was identified in 47% of AML cases, but was absent in myeloid cells in normal bone marrows. Our results suggest such combination therapies may significantly improve the therapeutic efficacy of FLT3 inhibitors. PRL-3 plays a potential pathological role in AML and it might be a useful therapeutic target in AML, and warrant clinical investigation.

## Introduction

Internal tandem duplication of fms-like tyrosine kinase 3 (FLT3-ITD) mutation occurs in about 25% of AML patients and is associated with poor prognosis [1], [2], [3]. In contrast to their impressive potency in cell culture system, current FLT3 inhibitors as single agent predominantly induce transient reduction of peripheral, but not bone marrow blasts in clinical trials [4]. Combination with other small molecule drugs represents a promising strategy to improve therapeutic efficacy of FLT3 inhibitors in clinic.

Histone acetylation and deacetylation, controlled by the balance of histone acetyltransferase (HAT) and histone deacetylase (HDAC), play a key role in regulating gene expression by changing chromatin structure. Small molecule HDAC inhibitors (HDACi) have proven to be a promising new class of anticancer drugs against hematological malignancies [5], as well as solid tumors [6]. Suberoylanilide hydroxamic acid (SAHA, Vorinostat®) is the first HDACi that obtained US FDA approval for the treatment of relapsed or refractory cutaneous T-cell lymphoma (CTCL).

SAHA has also been examined in a combinatory fashion with other classes of anticancer agents in acute leukemias. Combination of SAHA with cyclin-dependent kinase (CDK) inhibitor flavopiridol results in marked apoptosis through the downregulation of short-lived pro-survival proteins XIAP and Mcl-1 in AML cells [7]. Co-exposure of 17-allylamino- 17-demethoxygeldanamycin (17-AAG), a HSP90 antagonist, with SAHA induces profound mitochondrial damage and apoptosis through the inactivation of ERK activity and a block in p21^WAF1^ induction in leukemia cells [8]. Furthermore, inactivation of Akt and activation of c-Jun N-terminal kinase (JNK) has been identified as the mechanism of synergistic antileukemic effect between 2-medroxyestradiol (2-ME) and SAHA [9]. Specifically, HDAC inhibitors have been reported to synergistically interact with PKC412, a FLT3 inhibitor. LAQ824, a cinnamyl hydroxamate HDAC inhibitor, downregulates FLT3 receptor activity (p-FLT3) through disruption of chaperone protein HSP90, which stabilizes mutant FLT3 receptor [10], [11]. These data suggest that combination of HDAC inhibitors with different types of antitumor therapies might engage distinct molecules and signaling transduction pathways.

ABT-869, a multiple receptor tyrosine kinase inhibitor, inhibits FLT3 phosphorylation and signaling and is now in active clinical cancer therapeutic development [12]. In this study, we showed that combination of ABT-869 and SAHA has synergistic anti-leukemic activity. This study identified that PRL-3, a metastasis-associated gene, was a downstream target of FLT3-ITD signaling and played a role in the synergism. In addition, PRL-3 itself could be a new therapeutic target in AML.

## Results

### Synergistic cytotoxicity of combination of ABT-869 and SAHA in leukemia

**Figure 1:**
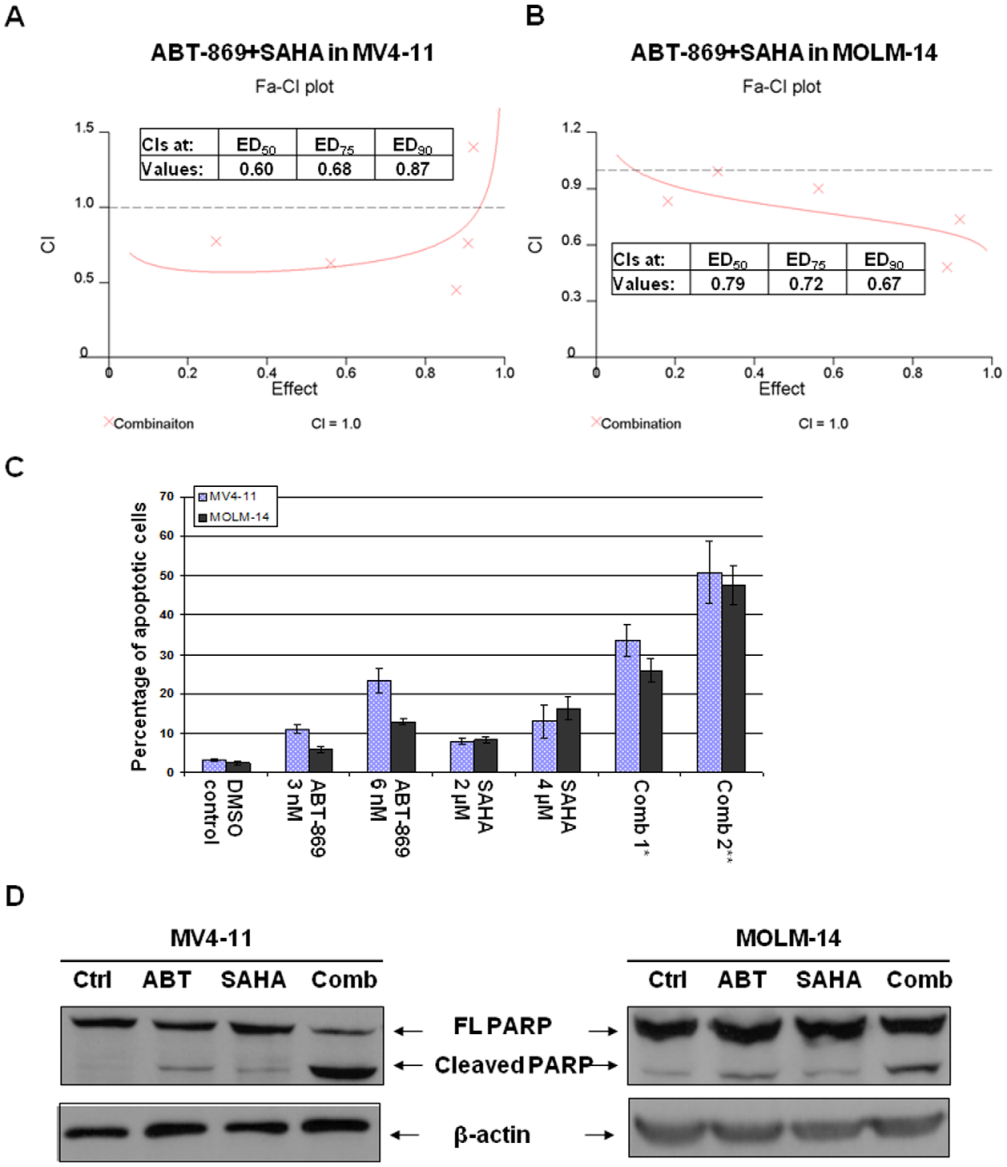
Antileukemic effect of the combination of ABT-869 with SAHA on leukemia cell lines with FLT3-ITD mutations. Combination indices (CIs) quantitatively describe the interactions between ABT-869 and SAHA in MV4-11 cells (A), MOLM-14 cells (B). The X-axis shows inhibitory effect by the combination of two drugs and Y-axis shows the CI values. ED stands for effective dosage. The CI values at ED_50_, ED_75_ and ED_90_ values of two drugs were inserted into the figures. These results were generated by CalcuSyn software. (C) Percentage of apoptosis induced by ABT-869 alone, SAHA alone, and combination treatment for 48 hours. The experiments were in triplicate. Comb 1*: Combination of ABT-869 3 nM and SAHA 2 µM, p<0.001 as compared to either single drug treatment. Comb 2**: ABT-869 6 nM+SAHA 4 µM, p<0.001 as compared to either single drug treatment. (D) Western blot analysis of cleaved PARP in MV4-11 and MOLM-14 cells. β-actin was used as loading control. Cells were treated with either control, ABT-869 3 nM for MV4-11 and 6 nM for MOLM-14, SAHA 4 µM or combination therapy for 48 hours, and then followed by lysis and Western blot analysis.

MV4-11 cells (M5) expressed exclusively the mutated allele of FLT3-ITD. MOLM-14 cells (M5) bear one allele of FLT3-ITD and the other allele of wild-type FLT3. We first determined the effect of HDACi on MV4-11 and MOLM-14 cells. Leukemia cell lines were treated with SAHA at increasing concentrations of 1 to 10 µM for 48 hours. MTS assays were used to determine the inhibition of cell proliferation. The ED_50_ of SAHA on MV4-11 and MOLM-14 was 4 µM and 5 µM respectively as determined by CALCUSYN software. Subsequently, we set about determining whether concurrent exposure of MV4-11 and MOLM-14 cells to ABT-869 and SAHA would result in enhanced cytotoxicity. As shown in Fig. 1A and 1B, the CI values at ED_50_, ED_75_ and ED_90_ ranged from 0.6 to 0.87, indicating synergistic effect.

To determine whether the combination therapy synergistically induce apoptosis, the Annexin-V/PI double staining was used to assess MV4-11 and MOLM-14 cells treated with ABT-869 and SAHA. Although exposure of MV4-11 and MOLM-14 cells to either ABT-869 or SAHA alone at indicated doses did not induce significant Annexin-V positive cells, the combination therapy demonstrated a marked increase in apoptosis in both cell lines (p<0.001, Fig. 1C). Importantly, individual drug exposure led to a modest expression of cleaved PARP, a hallmark of apoptosis. In contrast, co-treatment with ABT-869 and SAHA resulted in a remarked increase in cleaved PARP expression, indicating superior lethality (Fig. 1D).

These data therefore confirmed that combination of ABT-869 and SAHA resulted in significantly synergistic anti-leukemia effect in MV4-11 and MOLM-14 cells.

### ABT-869 and SAHA function synergistically to inhibit colony forming of AML cell lines and induce apoptosis in primary AML cells

**Figure 2:**
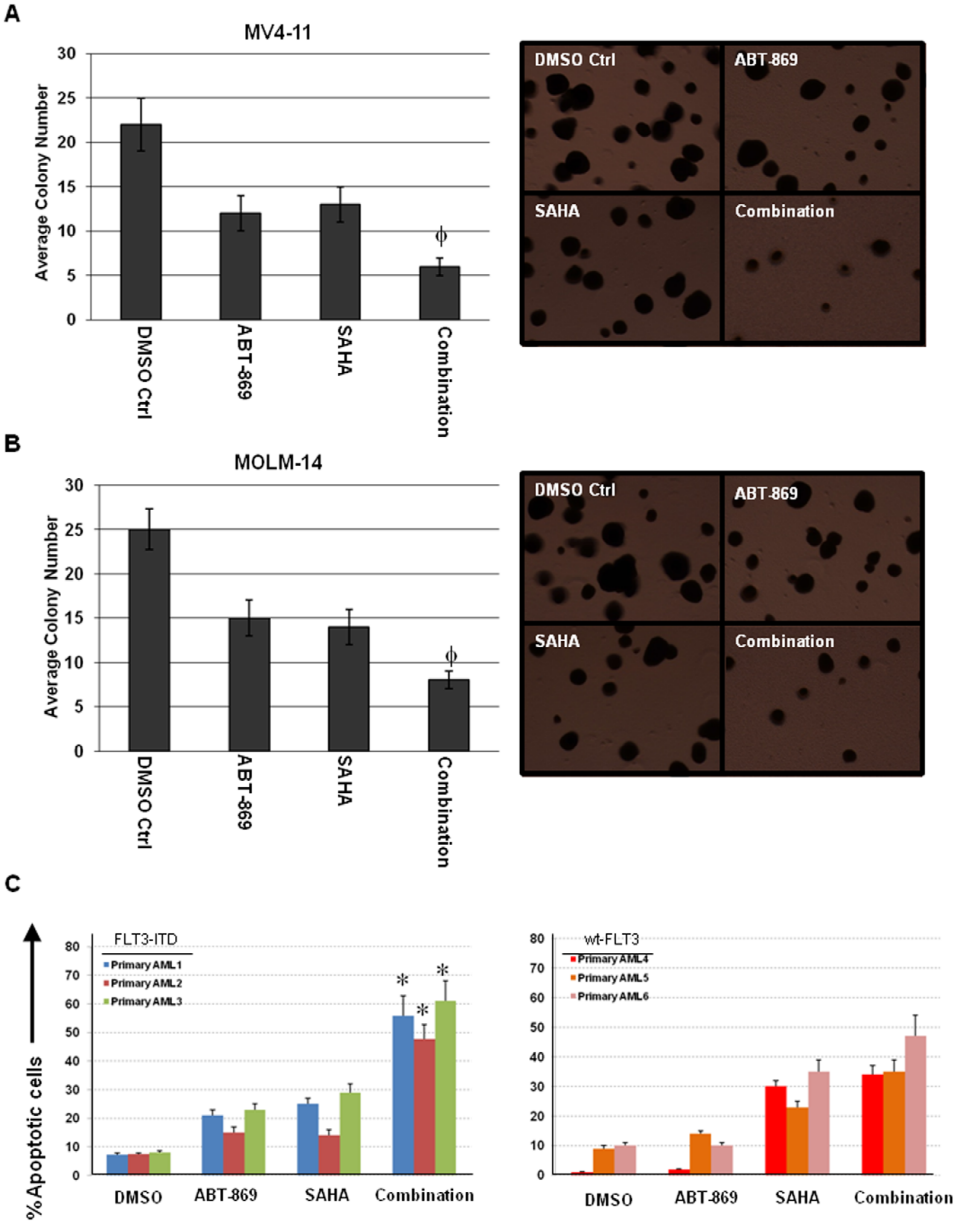
Combination treatment enhances loss of clonogenic survival and apoptosis of AML cells. (A) MV4-11 cells were treated with DMSO control, ABT-869 3 nM, SAHA 2 µM for 48 hours. (B) MOLM-14 cells were treated with DMSO control, ABT-869 6 nM, SAHA 2 µM for 48 hours. In both (A) and (B), cells were washed and plated in HSC-CFU media for 7 days. Representative pictures were presented in the right panel. The colony number was expressed as mean ± SD of average of 5 random 40 times fields. ^φ^p<0.01 as compared to single drug treatment. (C) Primary AML cells with FLT3-ITD mutation (left panel) or wt-FLT3 (right panel) were incubated with DMSO, ABT-869 20 nM, SAHA 4 µM and combination for 48 hours, followed by FACS analysis of apoptosis. The bar graphs show percentage of apoptotic cells. The experiments were duplicated. *p<0.001 as compared to single drug treatment in all the three primary AML patient samples with FLT3-ITD mutation.

Next, we investigated the cytotoxic effect of the combination treatment of ABT-869 and SAHA on clonogenic activity of MV4-11 and MOLM-14 cells. As shown in Fig. 2A and 2B, ABT-869 and SAHA alone moderately reduced the clonogenic activity of MV4-11 and MOLM-14. However, cotreatment with ABT-869 and SAHA not only significantly increased the loss of clonogenic survival of MV4-11 and MOLM-14 cells (p<0.01), but also decreased the size of the colonies in general, as compared to treatment with either of the drugs alone.

We further validated the findings by studying primary human leukemia. Primary cells from 3 patients with FLT3-ITD and 3 patients with wild type (wt) FLT3 were incubated with either DMSO, ABT-869 20 nM, SAHA 4 µM and in combination for 48 hours, followed by FACS analysis of Annexin V/PI double staining. Enhanced induction of apoptosis by combination treatment was observed in all the 3 primary AML samples with FLT3-ITD (all p values<0.001, Fig. 2C, left panel). However, the same combination treatment did not induced greater cell death as compared to SAHA alone in patients with wt-FLT3, while ABT-869 had little effect as expected (Fig. 2C, right panel).

### Identifying core gene signature crucial for the synergism between ABT-869 and SAHA

To elucidate the molecular mechanism of the synergistic lethality between ABT-869 and SAHA, we compared the gene expression profiles of MV4-11 and MOLM-14 cells treated with DMSO control, ABT-869, SAHA and combination therapy using the Affymetrix microarray platform. We focused on delineating a core gene signature unique and common to the combination therapy in both MV4-11 and MOLM-14, which could reveal important molecular insights into the therapeutic synergy we observed. Table 1 summarizes genes differentially induced more than two-fold by the combination therapy in both cell lines. The expression changes of some of the genes involved in cancer metastasis, cell cycle, DNA repair, DNA binding and cell proliferation, including Phosphatase of regenerating liver-3 (PRL-3, also named as PTP4A3), ORC1L, MND1, ZNF85 and LMO4 were confirmed by RQ-PCR at the mRNA level (Table S1 and Figure S1).

**Table 1:** List of core gene signature identified by Affymetrix microarray studies of MV4-11 and MOLM-14 cells treated with combination of ABT-869 and SAHA.

Probe ID	Gene Name	Description	Fold Change
1553743_at	FAM119A	family with sequence similarity 119, member A	−3.3
212975_at	DENND3	DENN/MADD domain containing 3	−2.3
209695_at	PTP4A3	protein tyrosine phosphatase type IVA, member 3	−2.5
205085_at	ORC1L	origin recognition complex, subunit 1-like (yeast)	−2.9
223700_at	MND1	meiotic nuclear divisions 1 homolog (S. cerevisiae)	−2.8
206572_x_at	ZNF85	zinc finger protein 85	−2.7
225362_at	FAM122B	family with sequence similarity 122B	−2.6
209608_s_at	ACAT2	acetyl-Coenzyme A acetyltransferase 2	−2.4
221750_at	HMGCS1	3-hydroxy-3-methylglutaryl-Coenzyme A synthase 1 (soluble)	−3.1
206632_s_at	APOBEC3B	apolipoprotein B mRNA editing enzyme, catalytic polypeptide-like 3B	−2.7
213008_at	KIAA1794	KIAA1794	−2.1
226817_at	DSC2	desmocollin 2	−2.5
214297_at	CSPG4	Chondroitin sulfate proteoglycan 4 (melanoma-associated)	−2.9
228385_at	DDX59	DEAD (Asp-Glu-Ala-Asp) box polypeptide 59	−2.1
1553972_a_at	CBS	cystathionine-beta-synthase	−2.5
226181_at	TUBE1	tubulin, epsilon 1	−2.4
1560023_x_at	---	CDNA FLJ37333 fis, clone BRAMY2020106	2.3
204072_s_at	FRY	furry homolog (Drosophila)	2.3
209205_s_at	LMO4	LIM domain only 4	2.2
228315_at	---	CDNA clone IMAGE:5261213	2.3
206332_s_at	IFI16	interferon, gamma-inducible protein 16	2.6
208966_x_at	IFI16	interferon, gamma-inducible protein 16	2.8
226030_at	ACADSB	acyl-Coenzyme A dehydrogenase, short/branched chain	2.3

Minus numbers denote the expression of the gene is decreased.

### Modulation of PRL-3 influenced drug sensitivity

**Figure 3:**
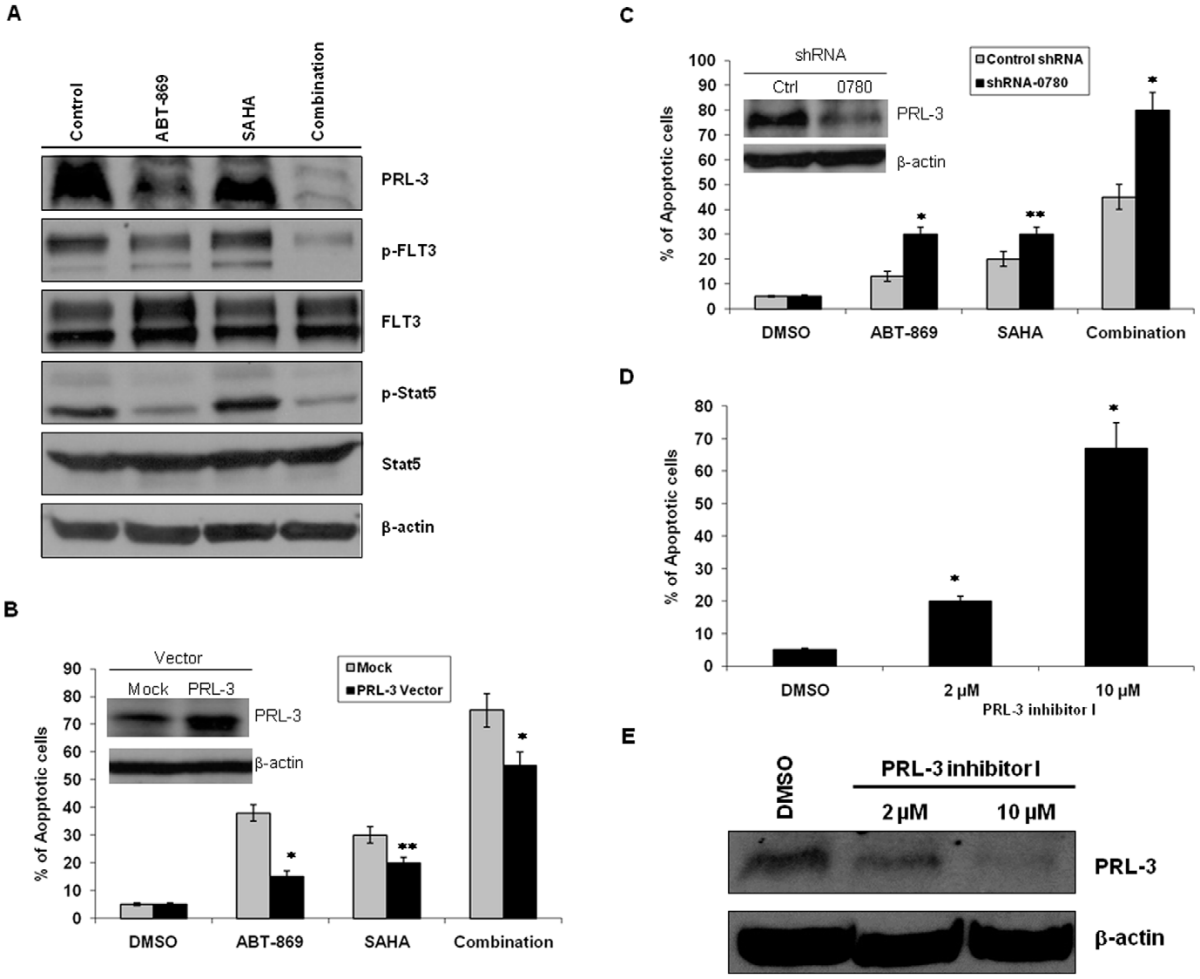
Impact of modulation of PRL-3 expression on drug sensitivity. (A) MOLM-14 cells were treated with DMSO, ABT-869 6 nM, SAHA 4 µM or combination therapy for 48 hours, and then followed by lyses and Western blot analysis of PRL-3, p-Stat5, Stat5. Immunoprecipitation (IP) was performed using anti-p-Tyrosine antibody, followed by Western blot with anti-FLT3 antibody. The same blot was then stripped and reprobed with anti-FLT3 antibody. The upper band is160 kDa glycosylated mature FLT3 and the lower band is 130 kDa nonglycosylated FLT3. (B) MOLM-14 cells were transfected with vector control (Mock) or pLVX-puro-PRL3 vector. Cells were treated with either ABT-869 6 nM, SAHA 4 µM or combination therapy for 48 hours. (C) MOLM-14 cells were transfected with control and PRL-3 shRNA-0780 respectively for 48 hours, and then treated with ABT-869 3 nM, SAHA 2 µM or combination therapy for 72 hours. Apoptosis assay was used to determine the cell viability in different treatments. (D) Flow cytomertric analysis of apoptosis in MOLM-14 cells were treated with either DMSO control or PRL-3 inhibitor I at concentration 2 and 10 µM for 48 h. (E) Effect of PRL-3 inhibitor I on PRL-3 protein expression. Apoptosis data shown represents the means of three independent experiments ± SD (*p<0.01, **p<0.05).

Among the top 5 downregulated genes, was PRL-3, a metastasis-associated gene, which has been shown to be oncogenic in several types of solid tumors. The finding that PRL-3 was significantly down-regulated by combination therapy prompted us to further explore the role of PRL-3 in the synergistic cytotoxicity. To investigate the effect of different treatment on PRL-3 protein expression, MOLM-14 cells were treated with control, ABT-869 6 nM, SAHA 4 µM, or combination. After 48 hours, cells were harvested for Western blot analysis. ABT-869 substantially reduced PRL3 protein and the combination therapy completely inhibited PRL-3 expression (Fig. 3A). To examine the role of FLT3 signaling in the synergism and regulation of PRL-3, we analyzed the expression of p-FLT3, FLT3 as well as p-Stat5, and Stat5, a downstream target of FLT3 pathway. In agreement with the changes on PRL-3, we observed the parallel change of p-FLT3, i.e., the inhibition was more profound in combination treated sample compared to ABT-869 alone (Fig. 3A). These data suggest that this synergistically anti-leukemic effect is FLT3 signaling-dependent.

Next, we established a PRL-3 over-expressing cell line, MOLM-14-PRL3 and the increased PRL-3 expression confirmed by Western blot analysis was 3-fold (Fig. 3B, inserted picture). Cells were treated with ABT-869, or SAHA at different concentrations, or in combination for 48 hr, followed by assessment of apoptosis. As shown in Fig. 3B, cells transduced with PRL-3 were more resistant not only to ABT-869 and SAHA as single agents, but also to the combination therapy, as compared with cells transduced with empty vector (p<0.05). Consistent with these results, the reduction of PRL-3 protein was also less in MOLM-14-PRL3 cells undergoing different treatments (Figure S2).

We next tested the effect of targeting PRL-3 on ABT-869-mediated cytotoxicity. We used shRNA approach to specifically knockdown PRL-3 in MOLM-14 cells, and then evaluated the cell viability after ABT-869 incubation by apoptosis assay. Among 3 different shRNA clones tested, RHS3939-98490780 (shRNA-0780) produced best knockdown effect. The approximately 90% downregulation of PRL-3 by shRNA-0780 was validated by Western blot analysis (Fig. 3C, inserted picture). We further observed that cells treated with PRL-3 shRNA-0780 were more sensitive to ABT-869, SAHA, and combination treatment as compared to control shRNA treated cells after 72 hours (Fig. 3C, p<0.05). In addition, we treated MOLM-14 cells with the small molecular PRL-3 inhibitor I, 2-Bromobenzyl-rhodanine derivative [13], for 48 h and observed increased portion of apoptotic cells (Fig. 3D, p<0.01) and reduction of PRL-3 protein in a dose-dependent manner (Fig. 3E).

**Figure 4:**
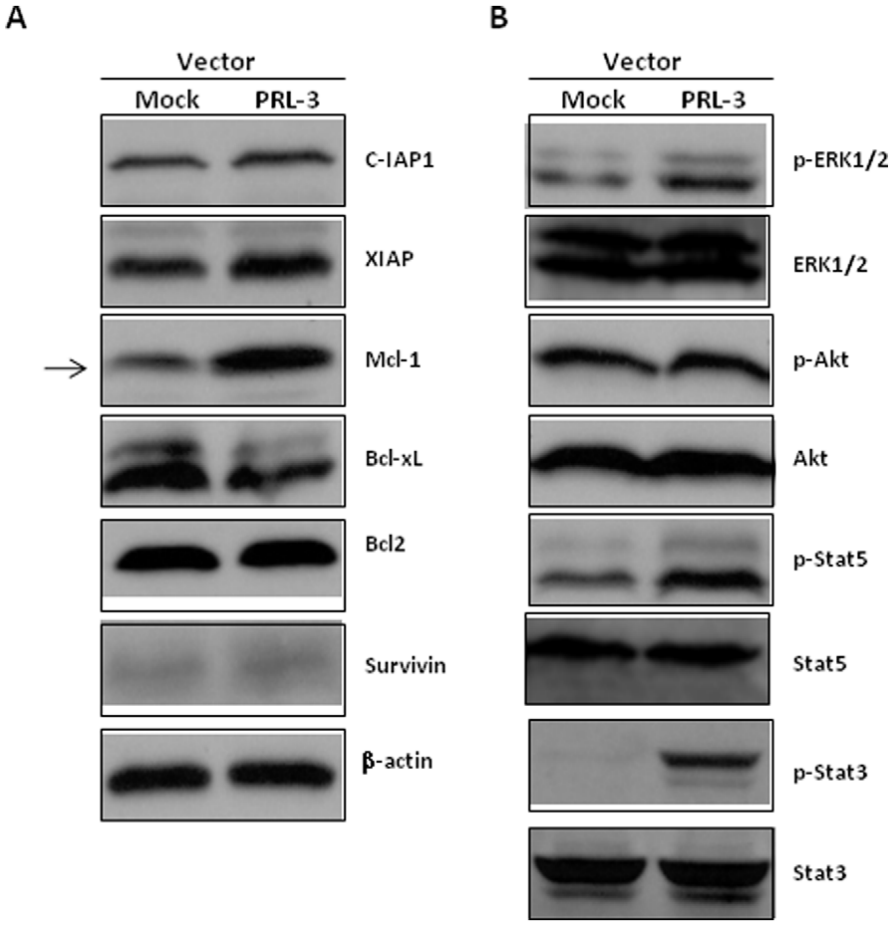
Comparisons of a panel of anti-apoptotic proteins and signaling transduction pathways in MOLM-14-Mock and MOLM-14-PRL3 cells. The two isogenic cells were lysed and subjected to 10% to 12% SDS-PAGE. Western blots were detected with the indicated antibodies for the assessment of expression level changes in anti-apoptotic proteins (A) and PI3K/Akt, MAPK/ERK and Stat pathways (B). β-actin was used as a loading control. Arrow highlighted Mcl-1.

To further investigate the molecular mechanisms underlying PRL-3 mediated drug resistance, we profiled a panel of anti-apoptotic proteins in MOLM-14-Mock and MOLM-14-PRL3 cells. Western blot analysis revealed that Mcl-1 was strikingly increased, followed by marginal upregulation of c-IAP1 and XIAP, while Bcl-xL, Bcl-2 and Survivin didn't increase in MOLM-14-PRL3 cells as compared to MOLM-14-Mock cells (Fig. 4A). Importantly, high level of Mcl-1 has been implicated in development and maintenance of both solid tumors and haematological malignancies and contributes to drug resistance [14]. Mcl-1 is regulated by several signalling pathways, including PI3K/Akt, MAPK/ERK and Stat pathways [14]. To that end, we further assessed the activities of the PI3K/Akt, MAPK/ERK and Stat pathways in these two isogenic cell lines. Interestingly, phosphorylated Stat5 (p-Stat5) and p-Stat3 were distinctly elevated, whereas p-ERK1/2 was minimally increased and there was virtually no change of p-Akt (Fig. 4B).

Taken together, these data demonstrate a role for PRL-3 in mediating drug resistance in AML through Stat activation and upregulation of Mcl-1, and the down-regulation of PRL-3 accounts for much of the synergistic effect between SAHA and ABT-869, which is dependent on FLT3 signaling.

### The association between PRL-3 expression and FLT3-ITD mutation in AML

**Figure 5:**
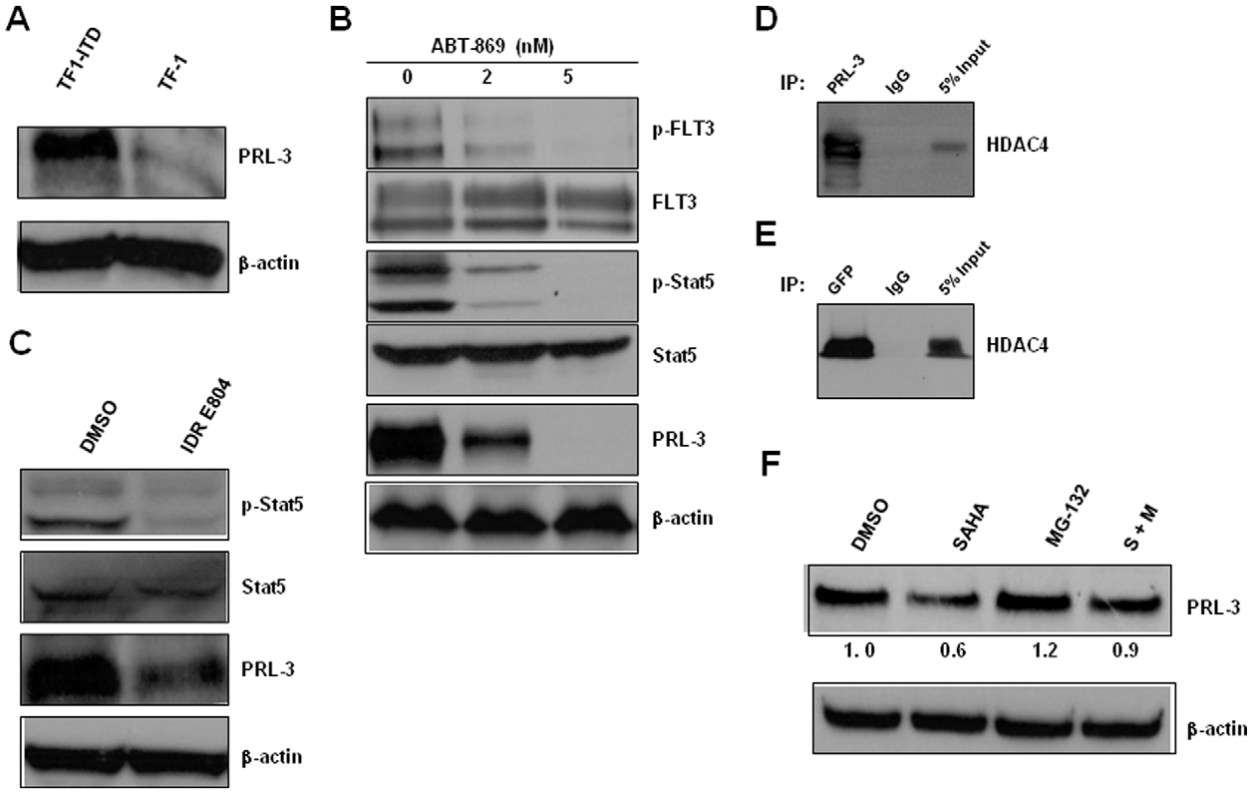
The association of PRL-3 and FLT3-ITD in AML. (A) Western blot assessment of PRL-3 protein in two isogenic cell lines, TF-1 and TF1-ITD. β-actin was used as loading control. (B) TF1-ITD cells were treated with DMSO, ABT-869 2 nM and 5 nM for 48 hours, and then followed by IP and Western blot analysis as described in Fig. 4.A. (C) MOLM-14 cells were treated with DMSO control or IDR E804 100 nM for 48 h. The cells were then lysed, and analysed by immunoblotting using anti-p-Stat5, anti-Stat5, anti-PRL-3 clone 318 and anti-β-actin. (D) MOLM-14 cell lysate was immunoprecipitated with anti-PRL-3 or anti-IgG antibody. The immunoprecipitates were immunoblotted with anti-HDAC4 antibody. (E) DLD-1-GFP-hPRL3 cell lysate was immunoprecipitated with anti-GFP or ant-IgG antibody, followed by immunoblotting analysis with anti-HDAC4 antibody. Total cellular extracts (inputs) were used as positive controls. (F) Western blot analysis of cell lysates extracted from MOLM-14 cells treated with DMSO, SAHA 4 µM, MG-132 10 µM or SAHA plus MG-132. The incubation duration of DMSO and SAHA was 24 h and MG-132 was added 4 h before harvest of cells. Densitometric analysis was performed using Amersham Image Scanner with LabScan ImageQuant TL Software. The level of PRL-3 was normalized with each β-actin level.

Oncomine is a web-based cancer microarray database, including 28000+ cancer transcriptome profiles. A search of the Oncomine database (December 09) revealed that PRL-3 was significantly overexpressed in FLT3-ITD positive AML as compared to FLT3-ITD negative AML (study name: Valk_leukemia, 78 vs 206 cases, p<0.001, Figure S3) [15], indicating a possible association between PRL-3 expression and FLT3-ITD mutation. Furthermore, when we examined the PRL-3 protein expression in two isogenic leukemia cell lines, TF-1 and TF1-ITD (stable cell line transfected with FLT3-ITD cDNA) [16], we found that the parental TF-1 cells were negative for PRL-3 protein. In sharp contrast, TF1-ITD showed abundant PRL-3 expression (Fig. 5A). To further validate the association between PRL-3 and FLT3, we treated the TF1-ITD cells with 2 nM and 5 nM of ABT-869. ABT-869 dose-dependently inhibited the expression of p-FLT3 and p-Stat5, as well as PRL-3 (Fig. 5B). We previously reported that indirubin derivative (IDR) E804, a specific Stat (Signal Transducer and Activator of Transcription) signaling inhibitor, reverses the resistance to FLT3 inhibitor in AML [12]. Here, we evaluated the role of Stat signaling in regulation of PRL-3. The data obtained showed the treatment with IDR E804 was effective in inhibiting Stat activity (p-Stat5) and reducing PRL-3 protein level (Fig. 5C). These results together with previous experiments showing that inhibition of PRL-3 by ABT-869 alone and combination treatment (Fig. 3.A) suggested PRL-3 could be a novel downstream molecule of FLT3-ITD signaling.

SAHA, a pan-HDAC inhibitor, targets both classes I and II HDAC enzymes [17]. To investigate the possible direct association of PRL-3 with HDACs, we undertook co-immunoprecipitation (Co-IP) of MOLM-14 cell lysate using anti-PRL-3 antibody. HDAC4 was present in immunoprecipiated PRL-3 complex, but not in the IgG control as shown in Fig. 5D. The interaction between HDAC4 and PRL-3 appeared specific, because Western blot analysis of HDAC1, 2, 3, 5, 6, 7 in the same immunoprecipiated complex turned out to be negative (data not shown). To further confirm the interaction between HDAC4 and PRL-3, PRL-3 complex were immunoprecipiated from cell lysate of colon cancer DLD-1 cells transfected with GFP-hPRL3 plasmid using anti-GFP antibody and screened for the presence of HDAC enzymes. Again, only HDAC4 was detected in the immunoprecipiated complex (Fig. 5.E and data not shown). These results demonstrated that HDAC4 bound to PRL-3 protein. To address the question of whether reduction of PRL-3 by SAHA was mediated through proteasome degradation, MOLM-14 cells were treated with SAHA, MG-132, a proteasome inhibitor, or SAHA plus MG-132. Western blot analysis showed the protein level of PRL-3 was decreased approximately 50% as compared to control sample, but this SAHA-induced inhibition of PRL-3 was blocked in the presence of proteasome inhibitor MG-132 (Fig. 5.F). Thus, these data implied that SAHA-related degradation of PRL-3 requires proteasome pathway.

**Figure 6:**
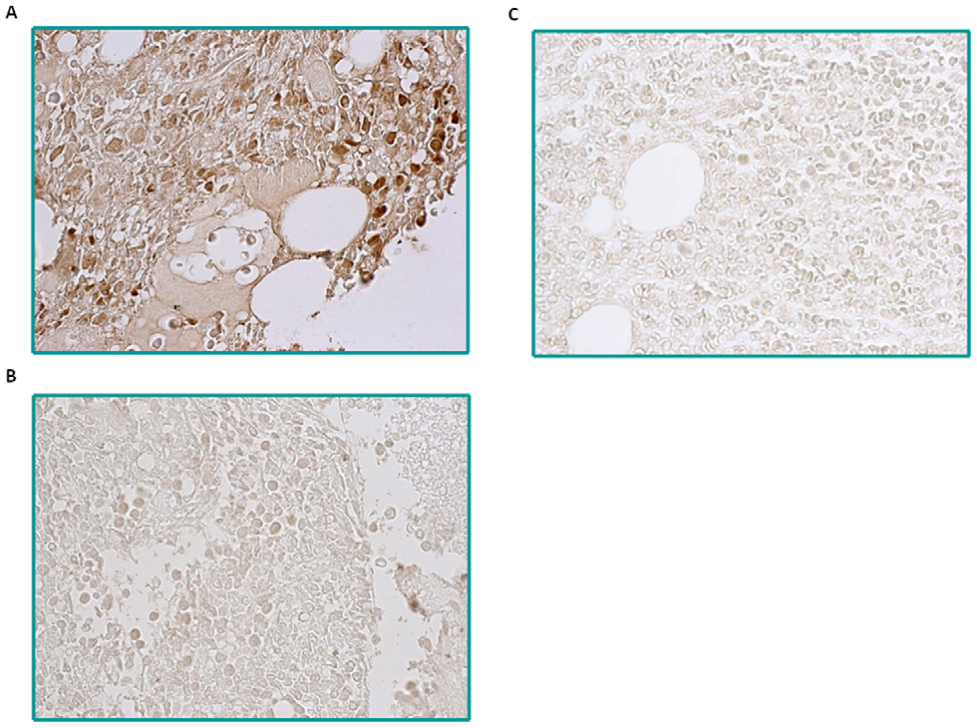
Evaluation of PRL-3 protein expression in bone marrow sections from primary AML patients and normal donors by IHC. (A) A representative image from nine PRL-3 positive AML cases was shown. PRL-3 positive signals were detected in the cytoplasm of myeloid cells. (B) A representative result from ten PRL-3 negative AML cases was shown. (C) A normal bone marrow image from 6 donors was presented. The original magnifications were 63×.

To further assess PRL-3 expression in patients with AML, we used the PRL-3-specific monoclonal antibody clone 318 to stain paraffin-embedded bone marrow sections of 19 newly diagnosed AML patients. We detected PRL-3 protein expression in 47% (9 out of 19) of AML samples (Fig. 6A and 6B), whereas PRL-3 expression is absent in 6 out 8 normal bone marrow samples (Fig. 6C). In 2 normal bone marrow samples, PRL-3 was detected in pre-erythrocytes, which is consistent with previous report from Guo K *et al* [18]. Among the PRL-3 positive samples, 67% (6 of 9) of patients are FLT3-ITD positive. This indicates PRL-3 could play a pathological role in leukemogenesis in AML, in particular, FLT3-ITD positive AML.

## Discussion

**Figure 7:**
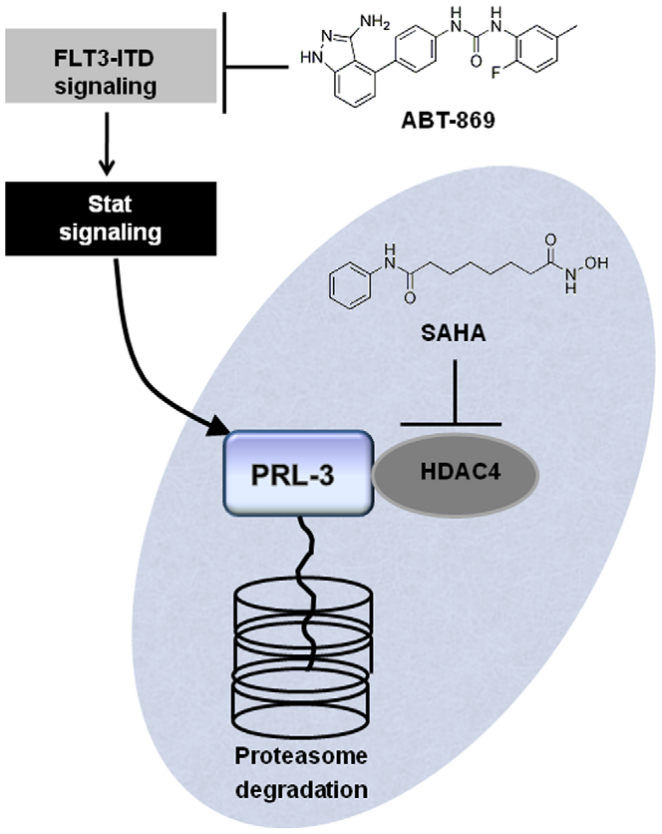
Schematic representation of the molecular mechanisms of enhanced downregulation of PRL-3 by combination treatment with ABT-869 and SAHA. ABT-868-induced inhibition of PRL-3 is due to targeting upstream FLT3-ITD and Stat pathway. HDAC4 physically interacts with PRL-3. SAHA mediated degradation of PRL-3 is dependent on the proteasome pathway.

We have demonstrated, in this study, the synergistic effect with the combination of ABT-869 and SAHA, a multi-targeted tyrosine kinase inhibitor and HDACi, a similar finding reported in a previous study using two related compounds [10]. However, we further have elucidated distinctive important downstream molecular mechanisms mediating this synergism, resulting in the identification of a novel signaling downstream of FLT3 involving PRL-3. Our data suggest a model in which the enhanced downregulation of PRL-3 is due to targeting two different pathways by ABT-869 and SAHA (Fig. 7). ABT-869 inhibits PRL-3 expression through suppression of FLT3-ITD and downregulated Stat pathways, while SAHA mediated degradation of PRL-3 is dependent on proteasome pathway (Fig. 7).

The PRL-3 gene encodes a 22-kDa tyrosine phosphatase that has been implicated in tumorigenesis and metastasis [19], [20]. Saha *et al.* reported exceptionally higher expression of PRL-3 in liver metastatic CRCs as compared to non-metastatic CRCs and normal colon epithelium [21]. Mechanistic studies reveal that PRL-3 functions as an initiator of neoplastic angiogenesis by recruiting endothelial cells [18] and stimulates invasion and motility of tumor cells through activating the Rho family of small GTPases such as RhoA and RhoC [22]. Increasing activities of Src kinase and PI3K/AKT signaling pathways via negative feedback regulation of C-terminal Src kinase (Csk) and PTEN tumor suppressor gene respectively by PRL-3 also contribute to its oncogenic role [23], [24], [25]. PRL-3 has been identified as a downstream target gene of p53 and it dose-dependently regulates cell-cycle progression [26]. Recently, we discovered that a trans-factor-PCBP1 (PolyC-RNA-binding protein 1) suppressed PRL-3 translation via binding three GC-motifs of the 5′ UTR of PRL-3 mRNA and an inverse correlation between PCBP-1 and PRL-3 protein levels in different solid tumors [27]. These findings highlight a fundamental role of PRL-3 in tumor development. In contrast to extensive studies in solid tumors, the role of PRL-3 in hematological malignancies is much less appreciated. To our knowledge, only one study reported that PRL-3 promotes human multiple myeloma (MM) cell migration and overexpression in a subset of MM patients assessed by gene expression profiling [28]. Herein, for the first time, we show that PRL-3 may play a role in drug resistance in AML and that modulation of PRL-3 expression plays a role in the synergistic antileukemic effect of co-treatment of ABT-869 and SAHA in FLT3-ITD positive AML. Importantly, our study uncovers a novel mechanism of PRL-3 mediated drug resistance by which PRL-3 activates Stat pathways and subsequently upregulates Mcl-1, conferring resistance to small molecule inhibitors. Higher level of Mcl-1 contributes to resistance to conventional chemotherapy and targeted therapies such as PKC412, another FLT3 inhibitor [29] and HDAC inhibitors [30], as well as ABT-737, a Bcl-2 antagonist [31]. Notably, in the present study, we demonstrate that PRL-3 may be a novel downstream target of FLT3-ITD signaling and expression of PRL-3 mRNA is significantly higher in AML patients with FLT3-ITD compared to wild type FLT-3. Furthermore, PRL-3 is aberrantly expressed in about half of AML cases, including 67% of FLT3-ITD positive AML samples in our study. The exact mechanism(s) by which some FLT3-ITD positive patients don't express PRL-3 protein remain elusive and are under further investigation. The full clinical implication of aberrant PRL-3 expression in AML will require study in a much larger patient population. Nevertheless, as PRL-3 is aberrantly expressed and pathologic relevance in a substantial number of AML, it may represent a good therapeutic target in this subset of AML. Developing small molecular inhibitor against phosphatases is currently an active area of research for cancer treatment [32]. In addition, monoclonal antibody targeting PRL-3 has recently been shown to inhibit metastasis of PRL-3-expressing cancer cells in murine xenograft models [33]. This clinical implication of PRL-3 targeted therapy in AML is under active investigation by our group.

In summary, we observed therapeutic synergism between ABT-869, a FLT3 and multi-targeted tyrosine kinase inhibitor, and SAHA, an HDAC inhibitor. We further elucidated the molecular basis for the synergism. In particular, our study highlights the critical role of PRL-3 in drug resistance and potential pathogenesis of AML. In addition, PRL-3 is aberrantly expressed in substantial number of AML, both with and without FLT3-ITD, suggesting that PRL-3 may have a significant role in leukemogenesis and could be a novel therapeutic target in PRL-3 positive AML.

## Materials and Methods

### Cell lines and primary patient samples

MV4-11, MOLM-14 and TF1-ITD cells were cultured with RPMI1640 (Invitrogen, Carlsbad, CA) supplemented with the addition of 10% of fetal bovine serum (FBS, JRH Bioscience Inc, Lenexa, KS) at density of 5×10^5^ cells/ml in a humid incubator with 5% CO_2_ at 37°C. TF-1 cells were cultured in RPMI1640 completed medium with 2 ng/ml human IL-3 (PeproTech, Rocky Hill, NJ). Bone marrow (BM) blast cells (>90%) from newly diagnosed AML patients were obtained at National University Hospital (NUH) in Singapore. The detection of FLT3 mutations was reported previously [34].

### Ethics Statement

This study was approved by Institutional Review Board (IRB) of National University of Singapore and BM samples were obtained with written informed consent.

### Drugs and chemicals

ABT-869 was kindly provided by Abbott Laboratories (Chicago, IL). ABT-869 was dissolved in dimethyl sulfoxide (DMSO) at concentration of 10 mM as stock kept in −20°C. SAHA was purchased from BIOMOL (Plymouth Meeting, PA). PRL-3 Inhibitor I (catalogue number P0108) and MG-132 were obtained from Sigma (St. Louis, MO) and dissolved in DMSO.

### Cell proliferation and apoptosis assays

Leukemic cells were seeded in 96-well culture plates at a density of 2×10^4^ viable cells/100 µl/well in triplicates, and were treated with ABT-869, SAHA or combination therapy. Colorimetric CellTiter 96 AQueous One Solution Cell Proliferation Assay (MTS assay, Promega, Madison, WI) was used to determine the cytotoxicity. IC_50_ was determined by MTS assay [12] and calculated with CalcuSyn software (Biosoft, Cambridge, UK). Each experiment was triplicated. For apoptosis assay, MV4-11 and MOLM-14 cells were cultured in the presence of either ABT-869, SAHA alone or in combination for 48 hours. Cells were washed twice with 1× Phosphate-Buffered Saline (PBS), stained with Annexin V/Propidium Iodide (PI, BD PharMingen, San Jose, CA), and immediately analyzed by a DakoCytomation Cyan LX flow cytometry (DakoCytomation, Glostrup, Denmark).

### Combination index calculation

The CalcuSyn software (Biosoft, Cambridge, UK) was used to analyze dose-effect relationships according to the method of Chou and Talalay [35]. The combination index (CI) was applied to quantitatively describe synergism defined as a greater-than-additive effect from a combination of two agents (CI<1), antagonism as a less-than-additive effect (CI>1), and an additive effect as (CI = 1). The CI values were calculated according to the levels of growth inhibition (Fraction affected, Fa) by each agent individually and combination of ABT-869 with SAHA. Specifically, the equation is: CI = (D)_1_/(D_x_)_1_+(D)_2_/(D_x_)_2_+(D)_1_(D)2/(D_x_)_1_(D_x_)_2_, where (D)_1_ indicates ABT-869 and D_2_ represents SAHA and D_x_ stands for the doses ABT-869 and SAHA alone, respectively, inhibiting x%. Constant ratio combinations of the two drugs at 0.25×, 0.5×, 1×, 2× and 4× of their ED_50_ was used.

### Western blot analysis

Preparation of the cell lysate and immunoblotting were performed as previously described [36], except for PRL-3 analysis, 16% gel was used. Anti-PRL3 antibody was clone 318 as reported previously [37].

### Co-immunoprecipitation (Co-IP) and immunoblotting

Anti-PRL-3 antibody clone 318 was attached to protein A/G agarose beads by using Pierce Crosslink IP Kit (Thermo Scientific, Rockford, IL) according to the manufacture's recommendation. Anti-GFP antibody conjugated agarose and anti-HDAC6 antibody were purchased from Santa Cruz biotechnology (Santa Cruz, CA). Histone Deacetylase (HDAC) Antibody Sampler Kit containing HDAC1, 2, 3, 4, 5, 7 was purchased from Cell Signaling Technologies (Danvers, MA). The establishment of the stable DLD-1 cell transfected with GFP-hPRL3 was reported previously (reference 32). Approximately 2×10^7^ MOLM-14 or DLD-1-GFP-hPRL3 cells were washed twice with ice-cold 1×PBS. Lysis was carried out on ice in NP-40 buffer (50 mM Tris-HCl, 150 mM NaCl, 1% NP-40, pH 8.0) containing Halt™ Protease inhibitor cocktail (EDTA-free) (Pierce, Thermo Scientific), phosphatase inhibitor cocktail I and II (Sigma-Aldrich, St Louis, MO, USA). The lysates were immunoprecipitated with the primary antibody overnight at 4, and the immunocomplexes were collected, washed four times with lysis buffer, and the pellet was resuspended in SDS sample buffer. Immunoprecipitates were boiled for 5 min, briefly centrifuged, and separated on 7.5% SDS-PAGE. Membranes were probed with the appropriate antibodies, and proteins recognized by the antibodies were detected using the Chemiluminescent Detection Kit (Pierece, Thermo Scientific).

### Colony forming capacity assay

MV4-11 and MOLM-14 cells were treated with ABT-869, SAHA or combination for 48 hours, then washed twice with 1×PBS. After that, about 3000 cells from control and drug-treated cells were plated in MACS® HSC-CFU media complete with Epo (Miltenyi Biotec GmbH, Germany) in 6-well plates and cultured for 7 days at 37°C in a 5% CO_2_ incubator. Colonies consisting of more than 50 cells were counted under an inverted microscope. Total 5 random 4×10 magnification fields were selected and average number of colonies of each sample was calculated. The experiments were duplicated.

### Microarray study

For the microarray experiments, MV4-11 and MOLM-14 cells were treated with DMSO control, ABT-869 3 nM, SAHA 6 µM and combination therapy for 24 hours. Cells were then washed in PBS and high-quality total RNA was extracted RNeasy Midi Kit, according to the manufacturer's instruction (Qiagen, Valencia, USA). RNA quantity, quality, and purity were assessed with the use of the RNA 6000 Nano assay on the Agilent 2100 Bioanalyzer (Agilent Technologies, Santa Clara CA, USA).

Gene expression profiling was performed using Affymetric U133plus2.0 gene chip (Affymetrix, Santa Clara, CA, USA) according to the manufacturer's protocol. Biological triplicates were performed for each sample. Three lists of genes with 2 fold or more difference in gene expression between treatment and control were generated as follows: ABT-869 alone versus DMSO control, SAHA alone versus DMSO control and ABT-869 plus SAHA versus DMSO control. This analysis was done separately for the MV4-11 and MOLM-14 cell lines and the final gene lists were genes that were unique to the ABT-869 and SAHA combination in both cell lines. Expression profiles were deposited into the Gene Expression Omnibus data repository (GSE accession # 26114) and are MIAME compliant.

### Construction and infection of PRL-3-expression vector

The human full-length cDNA of PRL-3 was purchased from Open Biosystems (Huntsville, AL) and inserted into *Eco*RI/*Bam*HI sites of lentivirus pLVX-puro vector (Clontech, Mountain View, CA), This pLVX-puro-PRL3 construct was validated by sequencing. Plasmid vectors were transfected into HEK 293T/17 packaging cells (ATCC) using Lentiphos™ HT protocol (Clontech, PT3984-2) as recommended by the manufacturer. High-titer viral particle-containing medium were harvested 48 h after transfection and used to infect MOLM-14 cells with 8 µg/mL polybrene (Millipore, Billerica, MA). Two days after infection, cells were transferred to fresh medium constituting 90% RPMI1640, 10% Tet System Approved FBS (Clontech) and 2 µg/mL puromycin (Millipore) for selection of transduced cells.

### Short-hairpin (shRNA) RNA study

Control and 3 human PRL-3 specifically pLKO.1 lentiviral shRNAs (RHS3939-9570404, -98490774 and -98490780) were purchased from Open Biosystems (Huntsville, AL, USA). Three million of MOLM-14 cells were mixed with viral supernatant and 8 µg/ml of polybrene (Millipore) and centrifuged at 2500 rpm for 90 min at 30°C. After additional incubation at 37°C for 4 hours, the medium was changed to fresh complete medium. Two days later, half of cells were harvested and followed by protein extraction and Western blot analysis of PRL-3 expression and the other half of cells were treated with different dose of ABT-869.

### Immunohistochemistry (IHC) analysis of primary AML bone marrow samples

AML patients with various genetic backgrounds as well as 3 normal controls were identified and bone marrow samples were retrieved from NUH tissue bank in accordance with the Declaration of Helsinki and the protocol was approved by the Institutional Review Board. Standard indirect immunoperoxidase procedures were used for immunohistochemistry with anti-PRL-3 specific antibody (clone 318) [37]. We used Dako EnVision™ Systems K 1395 (Dako, Carpinteria, CA) to perform IHC experiments.

## Supporting Information

Figure S1 **Real-time quantitative-PCR validation of some gene changes in the core gene signature identified by microarray studies.** (A) RQ-PCR quantification of PRL-3 gene. (B) RQ-PCR quantification of ORCL1 gene. (C) RQ-PCR quantification of MND1 gene. (D) RQ-PCR quantification of ZNF85 gene. (E) RQ-PCR quantification of LMO4 gene.
(DOC)

Figure S2 **Immunoblotting analysis of PRL-3 protein in MOLM-‵4 cells transfected with empty vector (Mock) or PRL-3 after treatment with either ABT-869 6 nM alone, SAHA 4 µM alone or combination therapy for 48 hours.** Beta-actin was used as loading control. Densitometric analysis was performed using Amersham Image Scanner with LabScan ImageQuant TL Software. The level of PRL-3 was normalized with respective β-actin level.
(DOC)

Figure S3 **The association of PRL-3 and FLT3-ITD in AML.** Comparison of PRL-3 expression between FLT3-ITD positive (Class 2) and others (Class 1, FLT3-ITD negative) AML patients. The box plot was generated by Oncomine based on the study of Valk P, *et al* (reference 15). The difference is statistically significant (p<0.001).
(DOC)

Table S1 **The sequences of primers used in real-time PCR.**
(DOC)
