# Supplementary material for: PRL-3, a Metastasis Associated Tyrosine Phosphatase, Is Involved in FLT3-ITD Signaling and Implicated in Anti-AML Therapy
Source: PLoS One. 2011 May 12;6(5):e19798. doi: 10.1371/journal.pone.0019798 (PMC3093398; doi:10.1371/journal.pone.0019798)
Supplement: Supplementary file 1 [file pone.0019798.s001.doc]

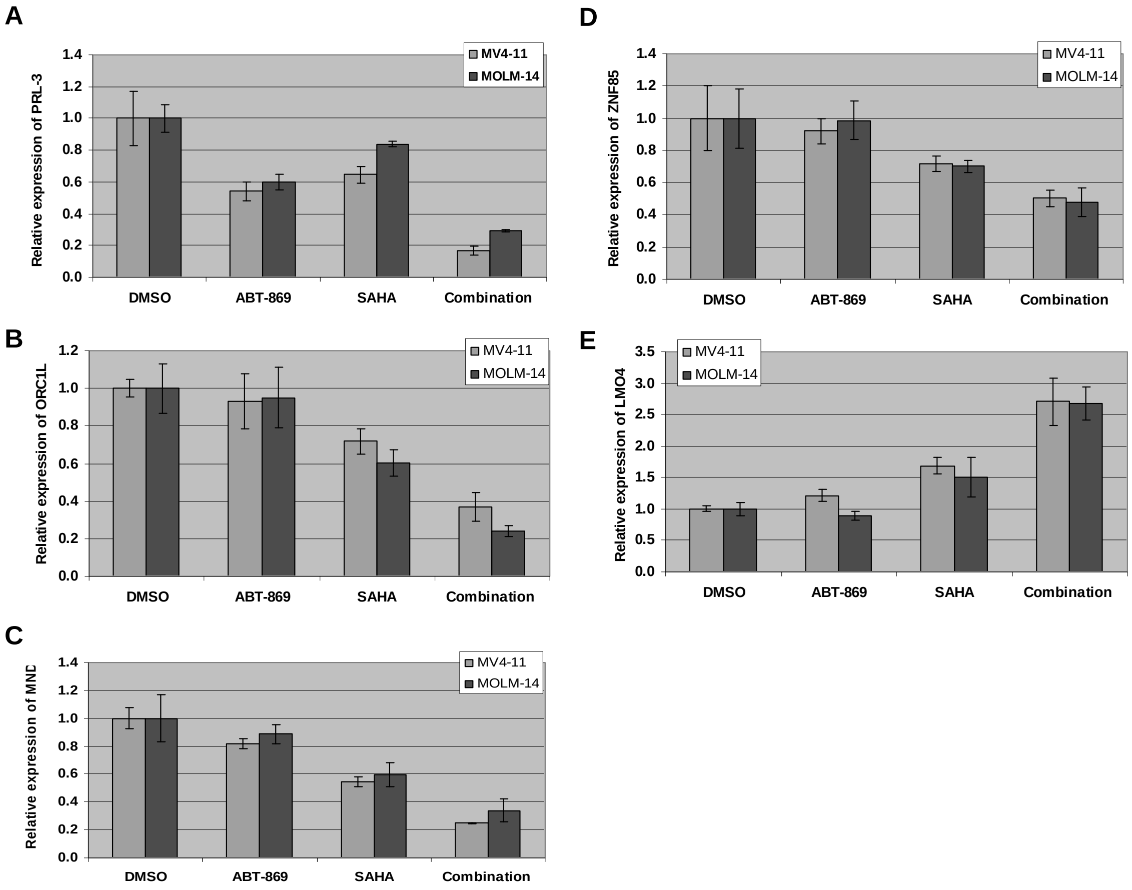
A
Relative expression of PRL-3
MV4-11
MOLM-14
DMSO
ABT-869
SAHA
Combination
B
Relative expression of ORC1L
MV4-11
MOLM-14
DMSO
ABT-869
SAHA
Combination
C
Relative expression of MN
MV4-11
MOLM-14
DMSO
ABT-869
SAHA
Combination
D
Relative expression of ZNF85
MV4-11
MOLM-14
DMSO
ABT-869
SAHA
Combination
E
Relative expression of LMO4
MV4-11
MOLM-14
DMSO
ABT-869
SAHA
Combination
