# Supplementary material for: PRL-3, a Metastasis Associated Tyrosine Phosphatase, Is Involved in FLT3-ITD Signaling and Implicated in Anti-AML Therapy
Source: PLoS One. 2011 May 12;6(5):e19798. doi: 10.1371/journal.pone.0019798 (PMC3093398; doi:10.1371/journal.pone.0019798)
Supplement: Supplementary file 2 [file pone.0019798.s002.doc]

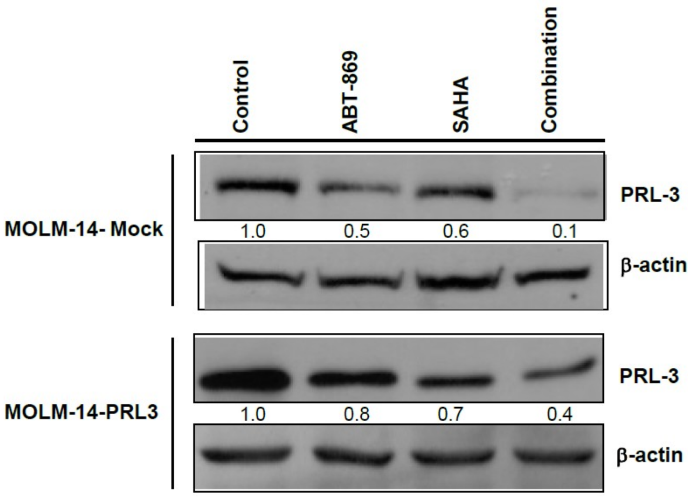
Control
ABT-869
SAHA
Combination
MOLM-14- Mock
PRL-3
1.0
0.5
0.6
0.1
β-actin
MOLM-14-PRL3
PRL-3
1.0
0.8
0.7
0.4
β-actin
