# Supplementary material for: PRL-3, a Metastasis Associated Tyrosine Phosphatase, Is Involved in FLT3-ITD Signaling and Implicated in Anti-AML Therapy
Source: PLoS One. 2011 May 12;6(5):e19798. doi: 10.1371/journal.pone.0019798 (PMC3093398; doi:10.1371/journal.pone.0019798)
Supplement: Supplementary file 3 [file pone.0019798.s003.doc]

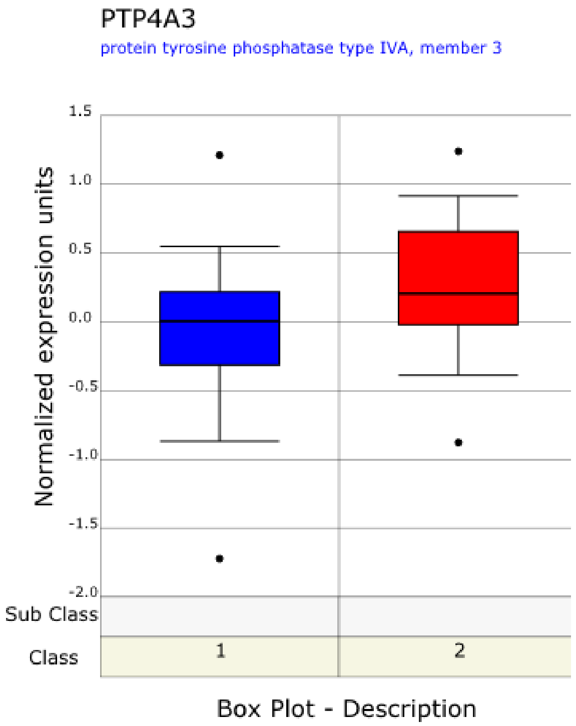
PTP4A3
protein tyrosine phosphatase type IVA, member 3
1.5
1.0
0.5
0.0
-0.5
-1.0
-1.5
-2.0
Normalized expression units
Sub Class
Class
1
2
Box Plot - Description
