# Supplementary material for: PRL-3, a Metastasis Associated Tyrosine Phosphatase, Is Involved in FLT3-ITD Signaling and Implicated in Anti-AML Therapy
Source: PLoS One. 2011 May 12;6(5):e19798. doi: 10.1371/journal.pone.0019798 (PMC3093398; doi:10.1371/journal.pone.0019798)
Supplement: Supplementary file 4 [file pone.0019798.s004.doc]

Gene	Forward Primer	Reverse Primer
PRL-3	5'-AGA AGG ATG GCA TCA CCG TTG T-3'	5'-ACT TCA TCC CGC TCT CAA TAA GCG-3'
ORC1L	5'-TTC TCG GAG ATC ACC TCA CCT TCT-3'	5'-AGCTGC AAT TCG GGT TCT CAG GAT-3'
ZNF85	5'-TAC AGA AAC CTG GTC TTC CTG GGT-3'	5'-ATA TTC TGC TCC GGC CAA AGG TCT-3'
MND1	5'-GGA GAA GAT TGC TCC CAA AGA GAA AGG C-3'	5'-TTC CGA TCC TCT CAC AGT CAA CCA-3'
LMO4	5'-GTC CCG GGA GAT CGG TTT CAC T-3'	5'-ATG GGA TCC ACC TGT GAT GAA CAA A-3'
β-Actin	5'-ATG TGG CCG AGG ACT TTG ATT-3'	5'-AGT GGG GTG GCT TTT AGG ATG-3'
